# Development of Orodispersible Tablets of Candesartan Cilexetil-***β***-cyclodextrin Complex

**DOI:** 10.1155/2013/583536

**Published:** 2013-09-24

**Authors:** Maddukuri Sravya, Rajamanickam Deveswaran, Srinivasan Bharath, Basappa Veerbadraiah Basavaraj, Varadharajan Madhavan

**Affiliations:** ^1^Department of Pharmaceutics, M.S. Ramaiah College of Pharmacy, Bangalore 54, India; ^2^Department of Pharmacognosy, M.S. Ramaiah College of Pharmacy, Bangalore 54, India

## Abstract

The aim of this study was to investigate the use of inclusion complexation technique employing *β*-cyclodextrin in improving the dissolution profile of candesartan cilexetil, a BCS class-II drug, and to formulate the inclusion complex into orodispersible tablets. The inclusion complexes were formed by physical mixing, kneading, coevaporation, and lyophilisation methods. Inclusion complexes were characterized by FTIR, DSC, XRD, NMR, and mass spectral studies. Inclusion complexes prepared using kneading, and lyophilisation techniques in the molar ratio 1 : 5 with *β*-cyclodextrin were used for formulating orodispersible tablets by direct compression with different superdisintegrants like croscarmellose sodium, crospovidone, sodium starch glycolate, and low substituted hydroxypropyl cellulose in varying concentrations. The directly compressible powder was evaluated for precompression parameters, and the prepared orodispersible tablets were evaluated for postcompression parameters. Drug-excipient compatibility studies showed no interaction, and characterization proved the formation of inclusion complex. *In vitro* disintegration time was found to be within 3 minutes, and all the formulations showed complete drug release of 100% within 20 minutes. The optimized formulation was found to be stable after 6 months and showed no significant change in drug content. This work proved *β*-cyclodextrins to be effective solubilizing agent in improving the solubility of poorly water soluble drugs.

## 1. Introduction

Any drug from a given dosage form to be absorbed must be present in the form of solution at the site of absorption. Low aqueous solubility is one of the major problems encountered during formulation development of new chemical entities especially in the process of generic product development. More than 40% of new chemical entities developed in pharmaceutical industry are practically insoluble in water. Various techniques are used for the enhancement of the solubility of poorly soluble drugs including physical and chemical modifications of drug like particle size reduction, crystal engineering, salt formation, solid dispersion, use of surfactant, hydrotropy, cosolvency, use of surfactants, and complexation [1]. Inclusion complexes are formed by the insertion of the nonpolar molecule or the nonpolar region of one molecule into the cavity of another molecule or host molecules. The most commonly used host molecules are cyclodextrins. Cyclodextrins are nonreducing, crystalline, water soluble, cyclic oligosaccharides consisting of glucose monomers arranged in a donut-shaped ring having hydrophobic cavity and hydrophilic outer surface. Three naturally occurring CDs are *α*-, *β*-, and *γ*-cyclodextrins. Cyclodextrins consist of six, seven, and eight D-glucose units, respectively, attached by *α*-1, 4-linkages. Cyclodextrins consist of (*α*-1,4)-linked *α*-D-glucopyranose units and contain a somewhat lipophilic central cavity and a hydrophilic outer surface. Due to the chair conformation of the glucopyranose units, the cyclodextrins are shaped like a truncated cone [2]. In aqueous solutions, cyclodextrins are able to form inclusion complexes with many drugs by taking up a drug molecule or more frequently some lipophilic moiety of the molecule, into the central cavity. No covalent bonds are formed or broken during the complex formation, and drug molecules in the complex are in rapid equilibrium with free molecules in the solution. 

For the inclusion complex formation, different methods were employed such as physical mixture, kneading, coevaporation, and lyophilisation techniques. The concept of mouth dissolving drug delivery system emerged from the desire to provide patient with more conventional means of taking their medication. It is difficult for many patients to swallow tablets. Hence, they do not comply with prescription, which results in high incidence of noncompliance and ineffective therapy. A mouth-dissolving drug delivery system, in most cases, is a tablet that dissolves or disintegrants in the oral cavity without the need of water or chewing [3]. Candesartan cilexetil is an angiotensin-II receptor antagonist used mainly for the treatment of hypertension. It is sparingly soluble in water belonging to BCS class-II, and its half-life is 5.1 h with oral bioavailability of 15% [4]. Therefore, in this present study, an attempt was made to formulate candesartan cilexetil, a poorly water soluble BCS class-II antihypertensive drug into an orodispersible tablet by forming an inclusion complex with an inert cyclic macromolecule *β*-cyclodextrins.

## 2. Materials and Methods

### 2.1. Materials

Candesartan cilexetil was a gift sample from Hetero Drugs Pvt. Ltd. (Hyderabad). *β*-Cyclodextrin (*β*-CD) was obtained from S D Fine-Chem. Ltd. (Mumbai, India). Crospovidone, croscarmellose sodium, and low substituted hydroxypropyl cellulose were obtained from Yarrow Chem. Products (Mumbai, India). Aspartame and aerosil was obtained from Himedia Laboratories Pvt. Ltd. (Mumbai, India). Mannitol was obtained from Thermo Fischer Scientific India Pvt. Ltd. (Mumbai, India). Sodium starch glycolate was obtained from E. Merck Ltd. (Mumbai, India). Methanol was obtained from RFCL Ltd. (New Delhi, India). All other ingredients used were of analytical grade.

### 2.2. Preparation of Cyclodextrin Inclusion Complexes [5]

#### 2.2.1. Physical Mixture

The physical mixture of candesartan cilexetil with *β*-CD was prepared by mixing candesartan cilexetil with *β*-CD in a mortar and pestle. Mixing was continued for one hour with constant trituration and then passed through sieve no. 100. The resulting sample was stored in a desiccator until further use. The physical mixture was prepared in 1 : 1, 1 : 3, and 1 : 5 molar ratios.

#### 2.2.2. Kneading Method

*β*-CD was taken in a mortar, and 10 mL of 50% ethanol was added and triturated to get slurry. Then, slowly drug was incorporated into the slurry, and trituration was further continued for one hour. Slurry was then air-dried at 25°C for 24 hours, pulverized, and passed through sieve no. 100. The resulting sample was stored in a desiccator until further use. Candesartan cilexetil complexes with *β*-CD were prepared in 1 : 1, 1 : 3, and 1 : 5 molar ratios.

#### 2.2.3. Coevaporation

Inclusion complex was prepared by dissolving 1 : 1, 1 : 3, and 1 : 5 molar ratios of *β*-CD and candesartan cilexetil in 10 mL of 50% aqueous ethanol. The solution was stirred till a clear solution was obtained, and the resulting solution was evaporated under vacuum at a temperature of 45°C. The solid residue was further dried completely at 45°C for 48 h. The dried complex was pulverized into a fine powder and sieved through sieve no. 100. The resulting sample was stored in a desiccator until further use.

#### 2.2.4. Freeze-Drying

Candesartan cilexetil and *β*-CD in molar quantities of 1 : 1, 1 : 3, and 1 : 5 were taken separately in 20 mL of water and mixed thoroughly using a magnetic stirrer. The resultant solution was frozen in a deep freezer at −20°C for about 12 h. The frozen mixture was then freeze-dried in the freeze-dryer for 8 h at −50°C under vacuum. The resultant product was stored in a desiccator.

### 2.3. Characterization of Complexes [6]

#### 2.3.1. Phase Solubility Studies

Phase solubility studies were carried out according to the method reported by Higuchi and Connors. An excess of candesartan cilexetil was added to 10 mL portions of distilled water, each containing variable amount of *β*-CD such as 0, 2, 4, 6, 8, and 10 milimoles/liter. All the solutions were shaken in rotary shaker for 72 hours. The solutions were filtered, and their absorbance was measured at 255.5 nm [7]. The apparent stability constants (1 : 1) were calculated from the phase solubility diagrams, according to the following equation:

(1) $$\begin{array}{c} K_{c}=\frac{\text{slope}}{S_{0}\left(1-\text{slope}\right)}, \end{array}$$

where *K*_*c*_ = apparent stability constant and *S*_0_ = intercept.

#### 2.3.2. Drug Content Estimation

100 mg of drug *β*-CD inclusion complex was accurately weighed and transferred to 100 mL volumetric flask. To this, 50 mL of 5% PEG 400 solution and 50 mL of distilled water were added. The resulting solution was diluted suitably, and drug content was estimated spectrophotometrically (Shimadzu, Japan, UV-1701) at 232 nm using distilled water as blank. The drug content was calculated using calibration curve.

#### 2.3.3. X-Ray Diffractometry (XRD)

The XRD patterns of drug, *β*-cyclodextrin, and complexes were recorded by using Philips Holland-PW 1710 scanner with filter Cu radiation over the interval 5–60°/2*θ*. The operation data were as follows: voltage 35 kV, current 20 mA, filter Cu, and scanning speed 1°/min.

#### 2.3.4. _1_H Nuclear Magnetic Resonance and Mass Spectroscopy Studies

_1_H nuclear magnetic resonance spectra were recorded on AMX400 NMR spectrometer. Chemical shifts (*δ*) are reported in parts per million downfield from internal reference standard tetramethylsilane [8]. Mass spectra were recorded on ESI-MS, Shimadzu, Japan, and molecular weight analysis was carried out [9].

#### 2.3.5. *In Vitro* Dissolution Studies

*In vitro* dissolution of candesartan inclusion complexes were studied in USP type II dissolution apparatus using 900 mL of distilled water as dissolution medium at 75 rpm and at a temperature of 37 ± 0.5°C. Complex equivalent to 8 mg of candesartan was used in each test. 5 mL of sample of dissolution medium was withdrawn at suitable time intervals, diluted suitably, and analyzed by measuring the absorbance at 255.5 nm. The volume withdrawn at each time interval was replaced with fresh quantity of dissolution medium to maintain sink conditions. The amount of candesartan released was calculated and plotted against time and compared with pure drug.

### 2.4. Formulation of Orodispersible Tablets of Candesartan-*β*-Cyclodextrin Complexes

Candesartan-*β*-cyclodextrin complex, mannitol, low substituted hydroxy propyl cellulose, crospovidone, croscarmellose sodium, sodium starch glycolate, aspartame, aerosol, and talc were weighed and passed through no. 60 mesh separately. Then, the ingredients were mixed thoroughly and compressed into tablets using 6 mm punches in rotary tablet press (Rimek RSB-4 Minipress, Cadmach). Formulations of candesartan cilexetil-*β*-cyclodextrin complex orodispersible tablets by direct compression method are shown in Table 1.

### 2.5. Precompression Parameters

The prepared granules were evaluated for angle of repose, bulk density, tapped density, Hausner's ratio, and Carr's compressibility index [10, 11].

### 2.6. Postcompression Parameters of Orodispersible Tablets [12]

#### 2.6.1. Weight Variation

Twenty tablets were selected at random and individually weighed, and the average weight was calculated. The uniformity of weight was determined according to pharmacopoeial specifications.

#### 2.6.2. Hardness and Friability

Hardness is the tensile strength of tablets expressed in kg/cm_2_, which was determined using Monsanto hardness tester. Preweighed sample of tablets was placed in the friabilator (Roche Friabilator) and operated for 100 revolutions. Tablets were dusted and reweighed. 

Percentage friability was calculated by using the formula

(2) $$\begin{array}{c} F=\frac{W_{\text{initial}}-W_{\text{final}}}{W_{\text{initial}}}\;\times 100, \end{array}$$

where *W*_initial_ = initial weight of 20 tablets and *W*_final_ = final weight of 20 tablets.

#### 2.6.3. Drug Content

Five tablets from each formulation were selected randomly, crushed and mixed. From the mixture powder equivalent to 10 mg of candesartan, cilexetil was weighed and dissolved in 100 mL of 5% PEG 400 in distilled water. The resulting solution was filtered through Whatman filter paper no. 41, diluted suitably, and the absorbance of the resulting solution was measured spectrophotometrically at 232 nm using distilled water as blank. Experiments were carried out thrice, and average percentage drug content was considered.

Drug content was estimated by the formula

(3) $$\begin{array}{c} \text{Drug}\;\text{content}=\frac{\text{concentration}\times \text{dilution}\;\text{factor}}{1000}, \end{array}$$

(4) $$\begin{array}{c} \%\;\text{Drug}\;\text{content}=\frac{\text{Practical}\;\text{yield}}{\text{Theoretical}\;\text{yield}}\times 100. \end{array}$$

#### 2.6.4. *In Vitro* Dispersion Time [13, 14]

*In vitro* dispersion time was measured by dropping tablets in a 10 mL measuring cylinder containing 6 mL of buffer solution simulating saliva fluid (pH 7.4). The time for the tablet to completely disintegrate into fine particles was noted. Six tablets from each formulation were randomly, selected and *In vitro* dispersion time was noted. 

#### 2.6.5. *In Vitro* Disintegration Time

The disintegration time for all formulations was carried out using tablet disintegration test apparatus. Six tablets were placed individually in each tube of disintegration test apparatus, and discs were placed. The water was maintained at a temperature of 37°  ± 2°C, and time taken for the entire tablet to disintegrate completely was noted.

#### 2.6.6. Wetting Time and Water Absorption Ratio

A tablet is placed on piece of tissue paper that was folded twice and kept in a petri dish (internal diameter = 6.5 cm) containing 6 mL of water, and the time for complete wetting is measured. The wetted tablet is then weighed, and the water absorption ratio, *R*, is determined using following equation:

(5) $$\begin{array}{c} R=\frac{100\left(W_{a}-W_{b}\right)}{W_{b}}, \end{array}$$

where *W*_*b*_ and *W*_*a*_ are the weights of tablet before and after water absorption, respectively.

#### 2.6.7. *In Vitro* Drug Release

*In vitro* drug release studies were carried out using 900 mL of distilled water as dissolution medium in USP dissolution apparatus type II rotating paddle apparatus. The rotating speed was maintained at 75 rpm, and temperature was maintained at 37°C ± 0.5°C. 5 mL of solution was withdrawn at regular predetermined time intervals, and same volume of sample was replaced with fresh dissolution medium. The samples were diluted suitable and the absorbances of the resulting solution were measured at 255.5 nm using UV/Visible spectrophotometer (UV-1601, Shimadzu, Japan). All the experiments were carried out in triplicate.

#### 2.6.8. Stability Study

The selected formulations were closely packed in aluminum foils and then stored at 40°C ± 2°C/75% RH ± 5% in stability chamber for 6 months and evaluated for their physical appearance, drug content, percent friability, and *in vitro *disintegration time at intervals of 2 months [15]. 

## 3. Results and Discussion

All the complexes were subjected to *in vitro* dissolution study, and complexes prepared by kneading method and freeze-drying method in the molar ratio of 1 : 5 were used for further study. The drug content of all the candesartan cilexetil-*β* cyclodextrin complexes was found to be 98.4%–99.9%. 

### 3.1. FTIR Studies

**Figure 1:**
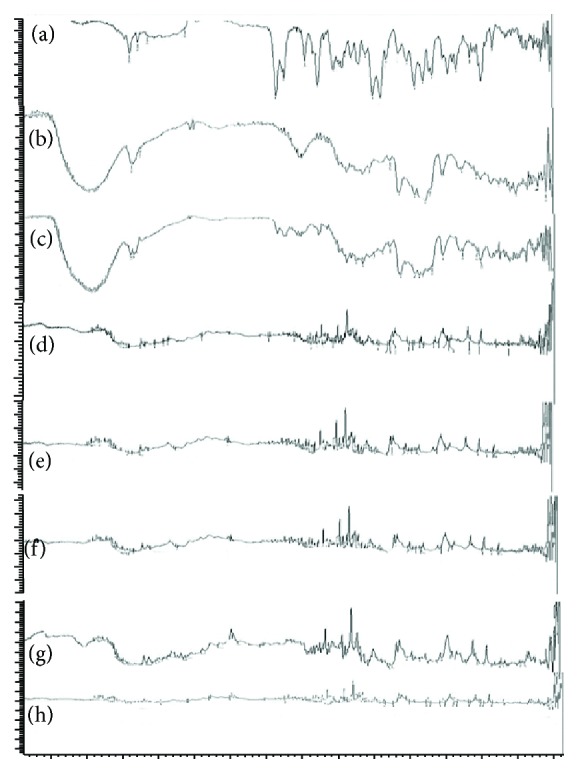
FTIR spectra. (a) FTIR spectra of candesartan cilexetil, (b) FTIR spectra of *β*-cyclodextrin, (c) FTIR spectrum of Candesartan cilexetil-*β*-cyclodextrin complex prepared by kneading technique (1 : 5 molar ratio of drug : *β*CD), (d) FTIR spectrum of Candesartan cilexetil-*β*-cyclodextrin complex prepared by lyophilisation technique (1 : 5 molar ratio of drug : *β*CD), (e) FTIR spectrum of Candesartan cilexetil-*β*-cyclodextrin freeze-dried complex with croscarmellose sodium, (f) FTIR spectrum of Candesartan cilexetil-*β*-cyclodextrin freeze-dried complex with crospovidone, (g) FTIR spectrum of Candesartan cilexetil-*β*-cyclodextrin freeze-dried complex with low substituted hydroxy propyl cellulose, and (h) FTIR spectrum of Candesartan cilexetil-*β*-cyclodextrin freeze-dried complex with sodium starch glycolate.

FTIR was performed for the pure drug *β*-cyclodextrin and its physical mixture to detect any sign of interaction which would be reflected by a change in the position or disappearance of any characteristic peaks of the compound. The IR spectra of the pure candesartan cilexetil (Figure 1) had shown characteristic peaks which are shown in Table 7. The IR spectra of the inclusion complexes prepared by kneading and lyophilisation methods showed the presence of characteristic peaks at the corresponding wave numbers of both pure candesartan cilexetil and *β*-cyclodextrin, thereby confirming the formation of an inclusion complex. The IR spectra of inclusion complexes along with the excipients used in the tablet formulation showed neither shift nor disappearance of characteristic peaks suggesting that there was no interaction between drug and *β*-cyclodextrin.

### 3.2. Differential Scanning Calorimetry

**Figure 2:**
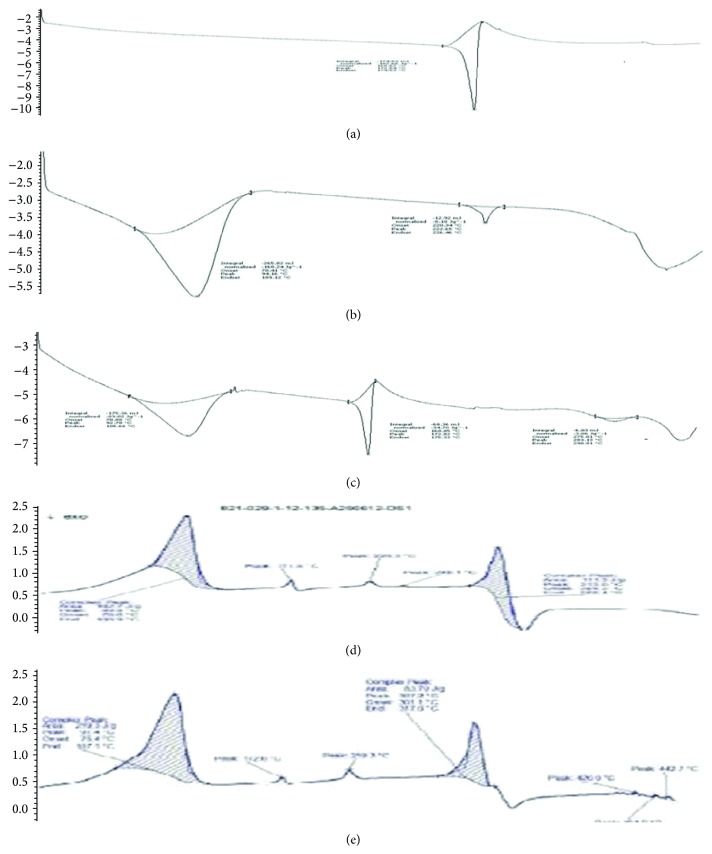
DSC thermograms. (a) DSC Thermogram of candesartan cilexetil, (b) DSC Thermogram of *β*-cyclodextrin, (c) DSC Thermogram of candesartan cilexetil with *β*-cyclodextrin (physical mixture), (d) DSC Thermogram of candesartan cilexetil with *β*-cyclodextrin (kneading method), and (e) DSC Thermogram of candesartan cilexetil with *β*-cyclodextrin (lyophilisation method).

One of the most classic applications of DSC analysis is the determination of the possible interactions between a drug entity and the excipients in its formulations. When guest molecules are included in CD cavities, their melting, boiling, glass transition, and sublimation points shift to different temperatures or disappear. Figures 2(a)–2(e) revealed the thermal behaviors of the pure components together with excipients. Candesartan cilexetil peaks are clear in its DSC thermogram (Figure 2(a)) demonstrating a sharp characteristic endothermic peak at 172.06°C, which is within its melting temperature range (*T*_*m*_); such endothermic peak signifies that candesartan cilexetil used was in pure crystalline state. *β*-Cyclodextrins peaks are distinct in Figure 2(b) demonstrating a broad characteristic endothermic peak at 222°C, which is within its melting range; such endothermic peak signifies that *β*-cyclodextrin was in pure crystalline state. DSC thermogram of the physical mixture of candesartan cilexetil with *β*-cyclodextrin (Figure 2(c)) demonstrated the presence of two endothermic peaks, both at melting range of candesartan cilexetil and *β*-cyclodextrin indicating not much interaction between them. DSC thermograms of inclusion complexes prepared by kneading and lyophilisation techniques (Figures 2(d) and 2(e)) showed distinct exothermic peaks at melting range of candesartan cilexetil and *β*-cyclodextrin, indicating the change in nature from crystalline to amorphous form. This change in nature from crystalline to amorphous form might be the reason for the enhanced solubility.

### 3.3. Characterization of Prepared Candesartan Cilexetil-*β*-Cyclodextrin Complexes

Inclusion complexes between candesartan cilexetil and *β*-cyclodextrin were prepared by physical mixture, kneading method, coprecipitation method, and lyophilisation method in the molar ratios of 1 : 1, 1 : 3, and 1 : 5. A total of 12 trial formulations has been prepared and evaluated for improvement in solubility of candesartan cilexetil. The most efficient preparation methods for inclusion complexes have been proven to be the lyophilisation method and kneading method, while the coprecipitation method is characterized by a lower reaction output due to the filtration step. All further studies have been performed on inclusion complexes prepared by lyophilisation method and kneading method in the molar ratio of 1 : 5 due to its improved solubility.

### 3.4. Phase Solubility Studies

**Figure 3:**
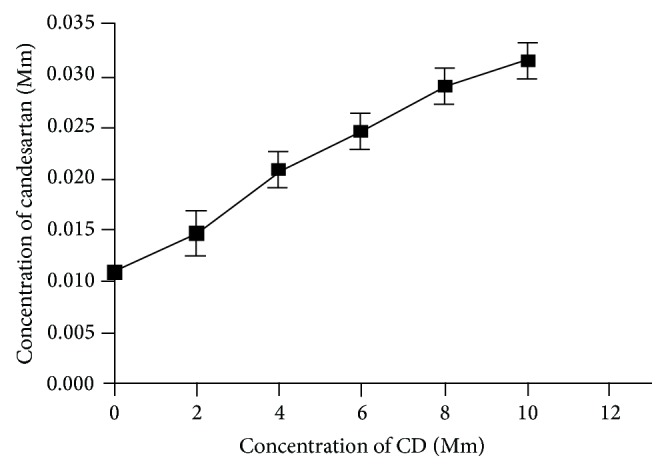
Phase solubility curve.

Analyzing the phase solubility profiles, it was observed that the increase of candesartan cilexetil solubility in the system was due to due to molecular interaction with *β*-CD (Figure 3). Candesartan cilexetil formed soluble complexes with *β*-CD in water showing a typical AL-type solubility diagrams where the regression coefficient of the curve and inclusion stability constant (*K*_*c*_) calculated according to equation were found to be 0.9867 and 0.04848 M^−1^. When the complex is first order with respect to ligand and first or higher order with respect to substrate, then AL-type phase solubility profile is obtained. The phase solubility diagram was classified as AL-type indicating the formation of a 1 : 1 candesartan cilexetil : *β*-CD inclusion complex. The lower values of the stability constants (*K*_*c*_) suggest that the candesartan cilexetil : *β*-CD interaction is weak and also that the inclusion process is exothermic, defined as enthalpically driven and spontaneous [16]. 

### 3.5. XRD Studies

**Figure 4:**
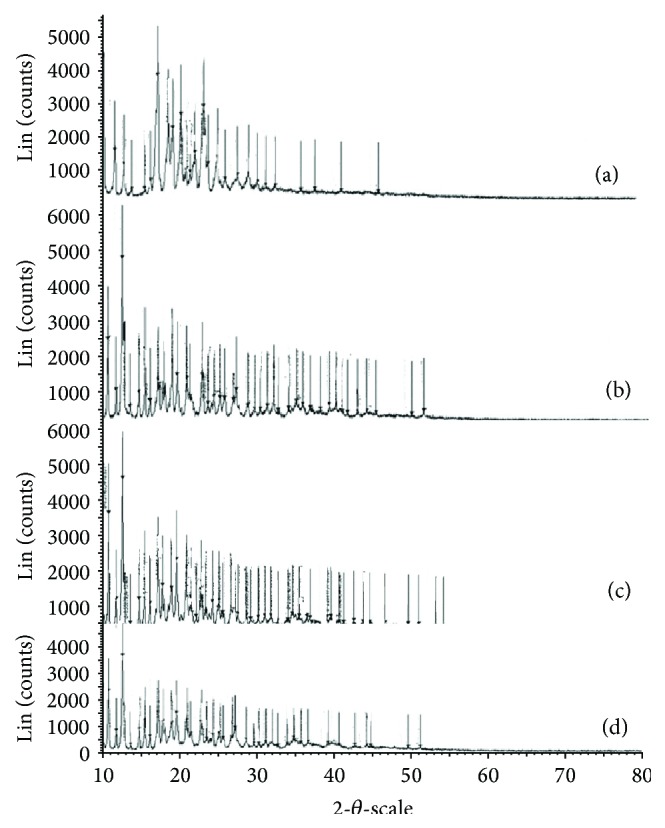
X-ray diffractograms. (a) X-ray diffractogram of candesartan cilexetil, (b) X-ray diffractogram of *β*-cyclodextrin, (c) X-ray diffractogram of candesartan cilexetil-*β*-cyclodextrin complex prepared by kneading method, and (d) X-ray diffractogram of candesartan cilexetil-*β*-cyclodextrin complex prepared by lyophilisation method.

The XRD results were in good agreement with the thermal analysis data. X-ray diffraction patterns in Figure 4(a) revealed that pure candesartan cilexetil was clearly in crystalline state as it showed sharp distinct peaks notably at 2*θ* diffraction angles of 9.97°, 17.04°, 18.74°, 19.40°, 21.63°, 23.94°, 25.16°, 27.96°, and 29.28°. X-ray diffraction pattern in Figure 4(b) revealed that pure *β*-cyclodextrin was in a crystalline state as it showed sharp distinct peaks at 2*θ* diffraction angles of 12.4° and 19.0° 2*θ*. X-ray diffraction pattern of inclusion complexes prepared by kneading and lyophilisation techniques (Figures 4(c)-4(d)) showed sharp distinct peaks at 2*θ* diffraction angles of 12.4°, 19.0°, 17.04°, 18.74°, and 19.40° that are characteristic for both candesartan cilexetil and *β*-cyclodextrin. The complex formation led to the broadening of the existing peaks, appearance of a few new peaks, and shifting of certain peaks. This could be due to the conversion of crystalline to amorphous form during complexation [17]. 

### 3.6. Mass Spectral Studies

**Figure 5:**
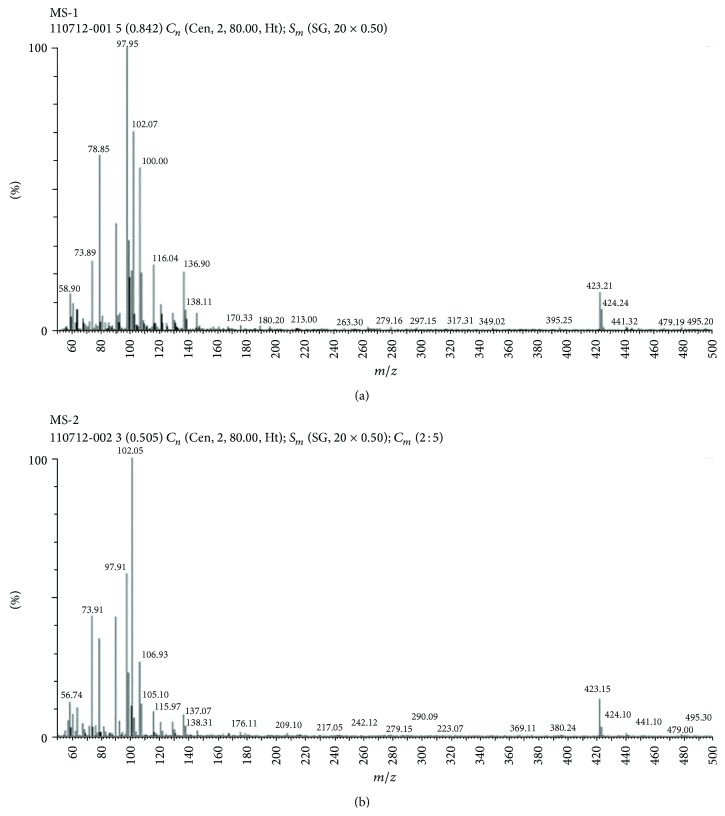
Mass spectral studies. (a) Mass spectrum of candesartan cilexetil-*β*-cyclodextrin complex prepared by kneading method, (b) mass spectrum of candesartan cilexetil-*β*-cyclodextrin complex prepared by lyophilisation method.

Mass spectroscopic studies were carried out to find out the molecular weight of candesartan cilexetil-*β*-cyclodextrin inclusion complexes prepared by kneading method and lyophilisation technique. From Figure 5(a), inclusion complex prepared by kneading method showed molecular ion peak at 102.05 and 423.18 on *m/z* scale. Other important fragments obtained were at 299 and 266 on *m/z* scale. From Figure 5(b), inclusion complex prepared by lyophilisation technique showed similar molecular ion peaks on *m/z* scale. Other important fragments obtained were at 299 and 148 on *m/z* scale. Extensive fragmentation seen in mass spectra may be attributed to large molecular weight of candesartan cilexetil and *β*-cyclodextrin. So based on the above, the molecular weight of the prepared complex was found to be 423.18 Dalton.

### 3.7. NMR Spectral Studies

**Figure 6:**
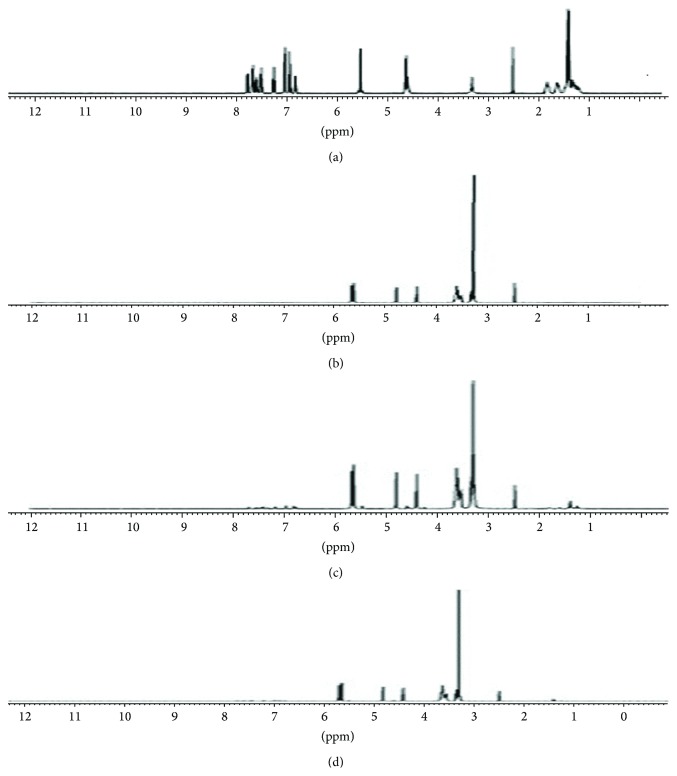
NMR spectra. (a) NMR spectra of candesartan cilexetil, (b) NMR spectra of *β*-cyclodextrin, (c) NMR spectra of candesartan cilexetil-*β*-cyclodextrin complex prepared by kneading method, and (d) NMR spectra of candesartan cilexetil-*β*-cyclodextrin complex prepared by lyophilisation method.

The _1_H-NMR chemical shift for each proton of candesartan cilexetil and *β*CD was evaluated for the formation of inclusion complex between candesartan cilexetil and *β*CD at molar ratio 1 : 5 of candesartan cilexetil and *β*CD. The chemical shifts for different protons in candesartan cilexetil (Figure 6(a)) were seen at 1-2, 2.4, 4.5, 5.5, and 6.8–7.6 ppm. The chemical shifts for different protons in *β*-cyclodextrins (Figure 6(b)) were seen at 2, 3.2, 3.6, 4.4, 4.8, and 5.8 ppm. The chemical shifts for the protons present in both candesartan cilexetil and *β*-cyclodextrins were found in inclusion complexes prepared by kneading method and lyophilisation technique (Figures 6(c)-6(d)). Since the complexes which were analysed were prepared using 1 : 5 molar ratio, the chemical shift values for protons present in candesartan cilexetil were seen in minimal intensity. These minimal intensity chemical shifts seen in inclusion complexes can also be accounted to the inclusion complexation of candesartan cilexetil into the cavity of *β*-cyclodextrins.

### 3.8. *In Vitro* Drug Release Profile

*In vitro* release studies were carried out using USP type II tablet dissolution test apparatus paddle method at 75 rpm and at a temperature of 37 ± 0.5°C, using 900 mL of distilled water as dissolution medium. Aliquots of 5 mL were withdrawn at a regular interval of 15 minutes and analyzed spectrophotometrically at 255.5 nm. The *in vitro* dissolution profiles of all the 12 complexes prepared by four different methods, namely, physical mixing, kneading, coevaporation, and lyophilisation techniques in three molar ratios of 1 : 1, 1 : 3, and 1 : 5, indicated faster drug release from all the formulations, and maximum drug release of 93.96% and 101.2% was obtained from formulations C6 and C12 that are prepared by kneading method and lyophilisation technique at the end of 105 min when compared to complexes prepared by coevaporation and physical mixture in all the other ratios.

### 3.9. Formulation of Candesartan Cilexetil-*β*-Cyclodextrin Orodispersible Tablets

Candesartan cilexetil-*β*-cyclodextrin inclusion complexes prepared by kneading method and lyophilisation technique at molar ratio 1 : 5 were selected for formulating into orodispersible tablets. Each complex was formulation into 12 formulations using four superdisintegrants, each of which is in three different concentrations as shown in Tables 1 and 2. 

### 3.10. Characterization of Prepared Orodispersible Tablet Formulation

#### 3.10.1. Flow Properties

The precompression parameters for the all 24 formulations were carried out, and the results were shown in Tables 3 and 4. The bulk density of all 24 formulations ranged between 0.361 and 0.421 g/mL, and tapped density ranged from 0.46 and 0.63 g/mL. Hausner's ratio with values less than 1.5 indicates good flow property. Carr's index value was between the range 16.7 and 35.6% which confirmed that all 24 formulations are showing good flow properties and good compressibility. The angle of repose was found to be from 18° to 31°, thereby confirming the good flow property of the granules. All the parameters were within the acceptable limits for powder blend with good flow properties while compressing the tablets.

#### 3.10.2. Weight Variation Test

The weights of all tablet formulations ranged between 137 and 161 mg (Table 5). As the weight of tablets was 150 mg, and the acceptable weight variation range was between 138.75 mg and 161.25 mg (±7.5%). It was observed that all the tablet formulations were within the pharmacopoeial limits.

#### 3.10.3. Hardness

The hardness of all formulations was determined, and the results were shown in Table 5. The values of hardness were found to be in the range of 4.2 to 5.8 kg/cm^2^.

#### 3.10.4. Friability

The percentage friability of all the formulations was found to be not more than 0.7%, which is well within the limit of less than 1%. The results of friability indicated that the tablets were mechanically stable.

#### 3.10.5. Drug Content

The drug content studies for all 24 formulations were carried out by a validated method using 5% PEG 400 in distilled water and was found to be in the range of 98.14%–100.28% of candesartan cilexetil, and the results were shown in Table 5.

#### 3.10.6. Wetting Time and Water Absorption Ratio

Wetting time of the formulations was determined, and all the formulations showed wetting time of 90 to 210 seconds. Water absorption ratio of all the formulations was calculated using the equation, and all the formulations showed good water absorption ratio from 72.58 to 81.42. Wetting time of the dosage form is related to contact angle. Lower wetting time implies a quicker disintegration time (Table 6).

#### 3.10.7. *In Vitro* Dispersion Time

*In vitro *dispersion time of all the formulations of orodispersible tablets was determined and was found to be 66 to 184 seconds. Formulations F_12_ and F_24_ showed good *in vitro* dispersion time of 94 and 66 seconds, respectively. 

#### 3.10.8. *In Vitro* Disintegration Time

*In vitro* disintegration time of formulations F_1_ to F_24_ was determined, and all the formulations showed disintegration time within three seconds. According to the European pharmacopoeia, the fast disintegrating or orodispersible tablets should disintegrate within 3 minutes without leaving any residue on the screen. 

#### 3.10.9. *In Vitro* Drug Release Profile

**Figure 7:**
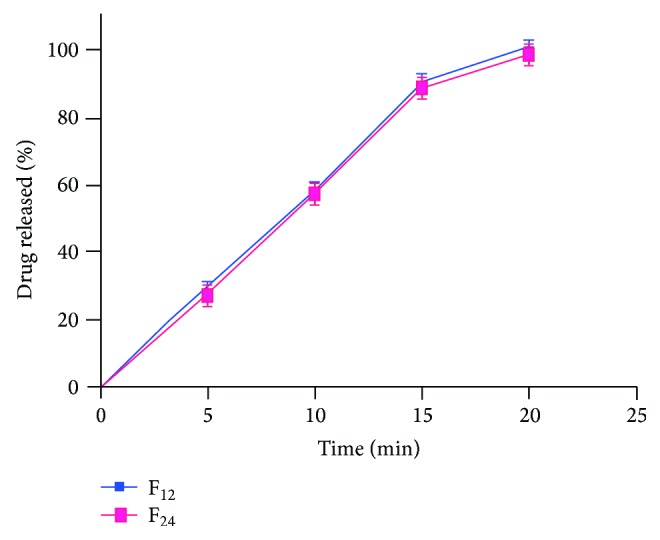
Graph showing drug release from formulations F_12_ and F_24_.

The prepared orodispersible tablets were evaluated for their *in vitro* drug release profiles. Formulations F_1_ to F_12_ were prepared using complexes prepared by kneading method, and formulations F_13_ to F_24_ were prepared using complexes prepared by lyophilisation method. Formulations containing low substituted hydroxyl propyl cellulose as superdisintegrant, namely, F_1_, F_2_, and F_3_, showed 97.8%, 100.01%, and 99.5%, respectively, at the end of 20 minutes, whereas formulations containing crospovidone as superdisintegrant, namely, F_4_, F_5_, and F_6_, showed 95.6%, 97.8%, and 98.9%, respectively, at the end of 20 minutes. Formulations containing croscarmellose sodium as superdisintegrant, namely, F_7_, F_8_, and F_9_, showed 97.7%, 98.8%, and 98.5%, respectively, at the end of 20 minutes, whereas formulations containing sodium starch glycolate as superdisintegrant, namely, F_10_, F_11_, and F_12_, showed 97.8%, 101.9%, and 101.2%, respectively, at the end of 20 minutes. Formulations containing low substituted hydroxyl propyl cellulose as superdisintegrant, namely, F_13_, F_14_, and F_15_, showed 95.6%, 96.7%, and 98.8%, respectively at the end of 20 minutes, whereas formulations containing crospovidone as superdisintegrant, namely, F_16_, F_17_, and F_18_, showed 97.8%, 99.9%, and 97.7%, respectively, at the end of 20 minutes. Formulations containing croscarmellose sodium as superdisintegrant, namely, F_19_, F_20_, and F_21_, showed 97.7%, 97.8%, and 98.8%, respectively, at the end of 20 minutes, whereas formulations containing sodium starch glycollate as superdisintegrant, namely, F_22_, F_23_, and F_24_, showed 98.9%, 100.9% and 101.1%, respectively at the end of 20 minutes. Formulations F_12_ and F_24_ prepared using cros povidone and sodium starch glycolate in the concentration of 8% showed lowest disintegration time of 74 and 68 seconds and highest drug release of 101.12 and 101.14 at the end of 20 minutes. The *In vitro *drug release study indicated faster and maximum drug release from formulations F_12_ and F_24_. The release profiles of formulations F_12_ and F_24_ were demonstrated in Figure 7.

### 3.11. Stability Studies

The accelerated stability studies of orodispersible tablets were performed as per the ICH guidelines to investigate whether the orodispersible tablets are affected during storage conditions. Optimized formulations F_12_ and F_24_ were kept at 40 ± 2°C with 75 ± 5% RH for a period of 6 months. The physical appearances, friability, disintegration time, and drug content were measured for these tablets at the end of 2, 4, and 6 months. The results showed that there was no significant difference between the initial and aged orodispersible tablets. The percentage drug content and friability were found to be 96.1 and 0.81% for formulation F_12_ and 96.3 and 0.88% for formulation F_24_ at the end of 6 months. This indicated that the physicochemical parameters, disintegration time, and percentage drug content were not affected by aging. The optimized formulations were physically and chemically stable for a period of 6 months at accelerated stability conditions. 

## 4. Conclusion

In the present study, complexes of candesartan cilexetil with *β*-cyclodextrins in molar ratio of 1 : 5 were prepared and characterized. These complexes were formulated into orodispersible tablets using varying concentrations of superdisintegrants. Precompression and postcompression parameters were determined, and all the parameters met the requirements. All the tablet formulations showed *In vitro *disintegration time of less than 3 minutes and 100 percent drug releases within 20 minutes. By this study, it can be concluded that *β*-cyclodextrins can be used as a solubilizing agent to improve solubility of poorly water soluble drugs. The oral bioavailability of the drug could be improved by this methodology by more than two times due to improved aqueous solubility when compared to pure drug. This methodology of inclusion complexation can be further exploited for the successful delivery of poorly water soluble compounds.

## Figures and Tables

**Table 1:** Formulation table for ODT's using complexes prepared by kneading method.

Formulations	Ingredients (mg) Total tablet weight = 150 mg								
	Candesartan-*β*-cyclodextrin complex	Mannitol	Low HPC	Crospovidone	Croscarmellose sodium	Sodium starch glycolate	Aspartame	Aerosil	Talc
F_1_	82	49	5	—	—	—	5	3	6
F_2_	82	46.5	7.5	—	—	—	5	3	6
F_3_	82	44	10	—	—	—	5	3	6
F_4_	82	49	—	5	—	—	5	3	6
F_5_	82	46.5	—	7.5	—	—	5	3	6
F_6_	82	44	—	10	—	—	5	3	6
F_7_	82	49	—	—	5	—	5	3	6
F_8_	82	46.5	—	—	7.5	—	5	3	6
F_9_	82	44	—	—	10	—	5	3	6
F_10_	82	49	—	—	—	5	5	3	6
F_11_	82	46.5	—	—	—	7.5	5	3	6
F_12_	82	44	—	—	—	10	5	3	6

**Table 2:** Formulation table for ODT's using complexes prepared by lyophilisation method.

Formulations	Candesartan-*β*-cyclodextrin complex	Mannitol	Low HPC	Crosspovidone	Cross carmellose sodium	Sodium starch glycolate	Aspartame	Aerosil	Talc
F_13_	82	49	5	—	—	—	5	3	6
F_14_	82	46.5	7.5	—	—	—	5	3	6
F_15_	82	44	10	—	—	—	5	3	6
F_16_	82	49	—	5	—	—	5	3	6
F_17_	82	46.5	—	7.5	—	—	5	3	6
F_18_	82	44	—	10	—	—	5	3	6
F_19_	82	49	—	—	5	—	5	3	6
F_20_	82	46.5	—	—	7.5	—	5	3	6
F_21_	82	44	—	—	10	—	5	3	6
F_22_	82	49	—	—	—	5	5	3	6
F_23_	82	46.5	—	—	—	7.5	5	3	6
F_24_	82	44	—	—	—	10	5	3	6

**Table 3:** Precompression parameters of prepared granules (F_1_ to F_12_).

Formulation code	Bulk density^a^ (g/mL)	Tapped density^a^ (g/mL)	Hausner's ratio^a^	Carr's index^a^ (%)	Angle of repose^a^ (*θ*)
F_1_	0.41 ± 0.002	0.61 ± 0.002	1.51 ± 0.002	33.31 ± 0.11	18°26′ ± 0.115
F_2_	0.41 ± 0.002	0.49 ± 0.002	1.25 ± 0.002	16.72 ± 0.15	22°58′ ± 0.642
F_3_	0.38 ± 0.002	0.54 ± 0.002	1.44 ± 0.002	30.64 ± 0.20	26°56′ ± 0.550
F_4_	0.41 ± 0.002	0.62 ± 0.002	1.50 ± 0.002	33.35 ± 0.11	25°64′ ± 1.155
F_5_	0.36 ± 0.002	0.51 ± 0.002	1.40 ± 0.01	28.61 ± 0.10	30°11′ ± 0.818
F_6_	0.36 ± 0.002	0.46 ± 0.002	1.27 ± 0.01	21.53 ± 0.05	18°26′ ± 0.346
F_7_	0.41 ± 0.002	0.55 ± 0.002	1.33 ± 0.011	25.04 ± 0.15	27°92′ ± 1.601
F_8_	0.42 ± 0.002	0.63 ± 0.002	1.52 ± 0.02	33.33 ± 0.20	22°78′ ± 4.215
F_9_	0.42 ± 0.002	0.56 ± 0.002	1.33 ± 0.01	25.08 ± 0.15	26°56′ ± 1.172
F_10_	0.38 ± 0.002	0.55 ± 0.002	1.44 ± 0.01	30.72 ± 0.15	27°47′ ± 0.692
F_11_	0.41 ± 0.002	0.54 ± 0.002	1.33 ± 0.01	24.91 ± 0.05	23°74′ ± 0.642
F_12_	0.41 ± 0.002	0.62 ± 0.002	1.50 ± 0.01	33.37 ± 0.15	22°78′ ± 0.692

^a^Mean ± S.D (*n* = 3).

**Table 4:** Precompression parameters of prepared granules (F_13_ to F_24_).

Formulation code	Bulk density^a^ (g/mL)	Tapped density^a^ (g/mL)	Hausner's ratio^a^	Carr's index^a^ (%)	Angle of repose^a^ (*θ*)
F_13_	0.41 ± 0.002	0.49 ± 0.002	1.25 ± 0.002	16.73 ± 0.11	22°58′ ± 0.115
F_14_	0.36 ± 0.002	0.56 ± 0.002	1.55 ± 0.002	35.64 ± 0.15	20°31′ ± 0.642
F_15_	0.42 ± 0.002	0.56 ± 0.002	1.33 ± 0.002	25.04 ± 0.20	21°80′ ± 0.550
F_16_	0.42 ± 0.002	0.56 ± 0.002	1.33 ± 0.002	25.08 ± 0.11	25°64′ ± 1.155
F_17_	0.38 ± 0.002	0.55 ± 0.002	1.44 ± 0.01	30.74 ± 0.10	30°11′ ± 0.818
F_18_	0.41 ± 0.002	0.54 ± 0.002	1.33 ± 0.01	24.92 ± 0.05	22°78′ ± 0.346
F_19_	0.41 ± 0.002	0.62 ± 0.002	1.50 ± 0.01	33.31 ± 0.15	23°26′ ± 1.601
F_20_	0.38 ± 0.002	0.54 ± 0.002	1.44 ± 0.02	30.65 ± 0.20	19°29′ ± 4.215
F_21_	0.36 ± 0.002	0.56 ± 0.002	1.55 ± 0.01	35.63 ± 0.15	27°47′ ± 1.172
F_22_	0.41 ± 0.002	0.62 ± 0.002	1.50 ± 0.01	33.34 ± 0.15	26°56′ ± 0.692
F_23_	0.42 ± 0.002	0.63 ± 0.002	1.53 ± 0.01	33.36 ± 0.05	18°77′ ± 0.642
F_24_	0.41 ± 0.002	0.54 ± 0.002	1.33 ± 0.01	24.90 ± 0.15	22°14′ ± 0.692

^a^Mean ± S.D (*n* = 3).

**Table 5:** Postcompression parameters for prepared orodispersible tablets.

Formulation code	Weight variation^a^ (mg)	Friability^a^ (%)	Hardness^a^ (kg/cm^2^)	Percentage drug content^a^
F_1_	140 ± 2.58	0.66 ± 0.01	5.1 ± 0.12	99.4 ± 0.68
F_2_	150 ± 2.65	0.67 ± 0.01	5.1 ± 0.05	99.8 ± 1.00
F_3_	150 ± 1.10	0.67 ± 0.01	5.2 ± 0.11	100.15 ± 1.00
F_4_	150 ± 2.58	0.66 ± 0.01	4.6 ± 0.11	98.66 ± 1.15
F_5_	150 ± 2.58	0.66 ± 0.01	5.2 ± 0.12	98.14 ± 1.15
F_6_	140 ± 2.66	0.67 ± 0.01	4.8 ± 0.05	99.71 ± 2.11
F_7_	154 ± 2.47	0.66 ± 0.05	5.5 ± 0.11	99.9 ± 0.92
F_8_	140 ± 2.70	0.66 ± 0.01	5.4 ± 0.05	100.15 ± 1.66
F_9_	156 ± 2.10	0.66 ± 0.02	5.2 ± 0.05	99.52 ± 1.15
F_10_	155 ± 4.10	0.67 ± 0.01	5.4 ± 0.01	100.28 ± 1.61
F_11_	157 ± 2.10	0.66 ± 0.05	4.2 ± 0.11	100.1 ± 0.69
F_12_	140 ± 4.10	0.66 ± 0.01	5.4 ± 0.01	98.66 ± 1.61
F_13_	150 ± 2.10	0.67 ± 0.05	4.7 ± 0.11	99.04 ± 1.61
F_14_	150 ± 2.45	0.66 ± 0.05	5.3 ± 0.16	99.23 ± 1.61
F_15_	140 ± 2.45	0.67 ± 0.01	5.3 ± 0.16	99.33 ± 1.89
F_16_	150 ± 2.67	0.66 ± 0.01	5.8 ± 0.19	99.71 ± 1.67
F_17_	158 ± 2.10	0.67 ± 0.01	5.4 ± 0.01	99.9 ± 1.61
F_18_	140 ± 4.10	0.66 ± 0.05	4.8 ± 0.11	100.3 ± 1.61
F_19_	150 ± 2.10	0.67 ± 0.01	5.5 ± 0.01	100.09 ± 1.89
F_20_	150 ± 2.45	0.66 ± 0.05	4.8 ± 0.11	100.28 ± 1.67
F_21_	140 ± 2.45	0.67 ± 0.05	5.6 ± 0.16	99.9 ± 1.15
F_22_	150 ± 2.67	0.66 ± 0.01	5.2 ± 0.16	99.71 ± 1.61
F_23_	150 ± 2.10	0.66 ± 0.01	5.8 ± 0.19	99.61 ± 0.69
F_24_	150 ± 2.45	0.67 ± 0.05	5.1 ± 0.19	99.52 ± 1.15

^a^Mean ± S.D (*n* = 3).

**Table 6:** Evaluation parameters of orodispersible tablets.

Formulation code	Wetting time (sec)^a^	Disintegration time (sec)^a^	*In vitro* dispersion time (sec)^a^	Water absorption ratio^a^
F_1_	180 ± 3	128 ± 4	172 ± 8	79.56 ± 0.6
F_2_	135 ± 4	114 ± 4	128 ± 6	79.83 ± 1.0
F_3_	120 ± 2	108 ± 3	122 ± 5	81.42 ± 1.0
F_4_	160 ± 5	105 ± 5	110 ± 6	75.52 ± 1.1
F_5_	144 ± 4	98 ± 6	102 ± 5	75.99 ± 1.1
F_6_	128 ± 3	85 ± 4	96 ± 6	76.05 ± 2.1
F_7_	210 ± 2	138 ± 4	182 ± 5	72.58 ± 0.9
F_8_	195 ± 4	124 ± 4	176 ± 4	73.14 ± 1.6
F_9_	184 ± 4	112 ± 5	166 ± 5	73.68 ± 1.1
F_10_	106 ± 4	98 ± 6	126 ± 6	79.18 ± 1.6
F_11_	100 ± 5	86 ± 3	106 ± 7	79.56 ± 0.6
F_12_	98 ± 3	74 ± 6	94 ± 6	80.08 ± 1.6
F_13_	172 ± 2	122 ± 5	150 ± 6	79.56 ± 1.6
F_14_	158 ± 4	110 ± 6	136 ± 6	79.83 ± 1.6
F_15_	152 ± 3	100 ± 5	124 ± 7	81.42 ± 1.8
F_16_	120 ± 4	95 ± 6	108 ± 6	75.08 ± 1.6
F_17_	108 ± 4	84 ± 5	98 ± 6	75.52 ± 1.6
F_18_	106 ± 3	76 ± 5	86 ± 5	75.99 ± 1.6
F_19_	190 ± 4	146 ± 6	172 ± 5	72.14 ± 1.8
F_20_	184 ± 5	114 ± 5	184 ± 4	72.86 ± 1.6
F_21_	162 ± 3	102 ± 6	168 ± 4	73.14 ± 1.1
F_22_	100 ± 3	90 ± 4	110 ± 5	79.18 ± 1.6
F_23_	92 ± 2	75 ± 5	84 ± 5	79.56 ± 0.6
F_24_	90 ± 4	68 ± 4	66 ± 4	80.08 ± 1.1

^a^Mean ± S.D (*n* = 3).

**Table 7:** IR spectral studies.

Sample	Wave number (cm^−1^)	Observation
Candesartan cilexetil	3083.38	C–H aromatic stretch
	3335.62	N–H secondary stretch
	1718.26	C=O stretch
	1248.86	C–N stretch and CH_3_ rock

*β*-Cyclodextrin	3612.12	–OH groups
	2934.34	CH_2_ stretch
	1205.21	Asymmetric C–O–C stretch

Inclusion complexes prepared by kneading method	3321.65	N–H secondary stretch
	3645.24	–OH groups
	1710.34	C=O stretch

Inclusion complexes prepared by lyophilisation	3346.87	N–H secondary stretch
	1705.46	C=O stretch
	2921.41	CH_2_ stretch
